# The Bacterial Proteasome at the Core of Diverse Degradation Pathways

**DOI:** 10.3389/fmolb.2019.00023

**Published:** 2019-04-09

**Authors:** Andreas U. Müller, Eilika Weber-Ban

**Affiliations:** Department of Biology, Institute of Molecular Biology and Biophysics, ETH Zurich, Zurich, Switzerland

**Keywords:** bacterial proteasome, pupylation, degradation, mycobacterial proteasomal ATPase Mpa, bacterial proteasome activator Bpa, Cdc48-like protein of actinobacteria Cpa

## Abstract

Proteasomal protein degradation exists in mycobacteria and other actinobacteria, and expands their repertoire of compartmentalizing protein degradation pathways beyond the usual bacterial types. A product of horizontal gene transfer, bacterial proteasomes have evolved to support the organism's survival under challenging environmental conditions like nutrient starvation and physical or chemical stresses. Like the eukaryotic 20S proteasome, the bacterial core particle is gated and must associate with a regulator complex to form a fully active protease capable of recruiting and internalizing substrate proteins. By association with diverse regulator complexes that employ different recruitment strategies, the bacterial 20S core particle is able to act in different cellular degradation pathways. In association with the mycobacterial proteasomal ATPase Mpa, the proteasome degrades substrates post-translationally modified with prokaryotic, ubiquitin-like protein Pup in a process called pupylation. Upon interaction with the ATP-independent bacterial proteasome activator Bpa, poorly structured substrates are recruited for proteasomal degradation. A potential third degradation route might employ a Cdc48-like protein of actinobacteria (Cpa), for which interaction with the 20S core was recently demonstrated but no degradation substrates have been identified yet. The alternative interaction partners and wide range of substrate proteins suggest that the bacterial proteasome is a modular, functionally flexible and conditionally regulated degradation machine in bacteria that encounter rapidly changing and challenging conditions.

## Introduction

The existence of a bacterial 20S proteasomal complex was reported for the first time for the nitrogen-fixing actinomycete *Frankia* (Benoist et al., 1992). Genetic and biochemical studies of the 20S proteasome in *Rhodococcus erythropolis* (Tamura et al., 1995; Nagy et al., 1997), *Mycobacterium smegmatis* (Knipfer and Shrader, 1997) and *Streptomyces coelicolor* (Nagy et al., 1998) soon followed, establishing its principal composition of homoheptameric α- and β-rings arranged in a stacked complex with two inner β-rings flanked by an α-ring on each side, as previously observed for archaeal and eukaryotic 20S proteasomes. From increasingly available sequenced bacterial genomes it became clear that proteasomes are an unusual occurrence in bacteria restricted to actinobacteria (De Mot et al., 1999). It has been surmised from eukaryote-typic features of the bacterial proteasomes (assembly, inhibitor profiles, β-subunit prosequence lengths) that they were obtained by horizontal gene transfer between a eukaryotic organism and an ancient actinobacterium after the split from other Gram-positive bacteria (Lupas et al., 1997). A divergent member of the AAA+ protein family was found to be encoded upstream of the proteasomal subunit genes forming part of this conserved gene locus (Wolf et al., 1998). Reminiscent of the ATPase subunits in the eukaryotic 19S regulatory particle, this AAA+ protein referred to as ARC (AAA protein forming ring-shaped complexes) features N-terminal coiled-coil domains followed by oligonucleotide binding domains, suggesting it, like the 20S subunits, might have been acquired by horizontal gene transfer from a eukaryotic organism (Djuranovic et al., 2009).

**Figure 1:**
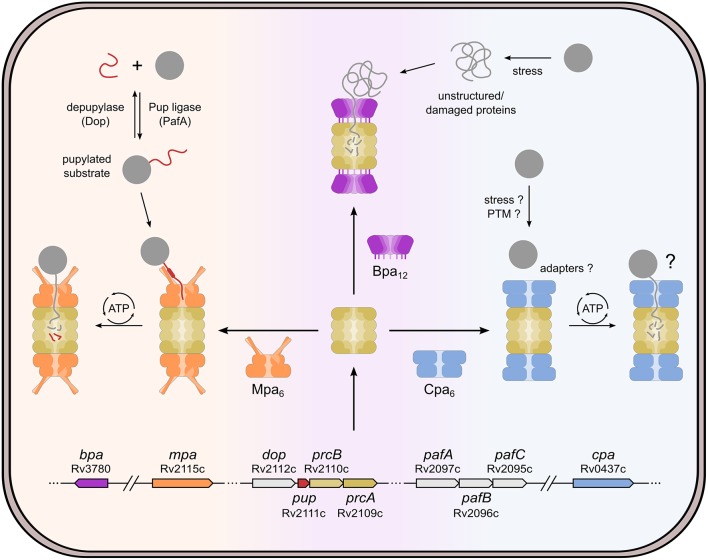
Cellular pathways involving the bacterial proteasome in *Mycobacterium tuberculosis*. The proteasome core particle (beige) can interact with different regulator complexes: Mpa (mycobacterial proteasome activator, orange), Bpa (bacterial proteasome activator, purple) or Cpa (Cdc48-like protein of actinobacteria, blue). By formation of different activator-proteasome complexes the cell can tune the degradation activity according to its needs under different conditions. The Mpa-proteasome degrades otherwise stable, folded proteins that have been tagged with Pup (prokaryotic ubiquitin-like protein, red). The Pup-ligase PafA, depupylase Dop, Pup, Mpa, and the proteasome subunits are all encoded in close proximity to one another in a region of the genome referred to as Pup-proteasome system (PPS) gene locus. Pupylation-mediated degradation was shown to be important under various conditions, such as recovery from DNA damage, nitrogen starvation, and persistence in the host macrophage. Bpa forms a dodecameric ring with a large pore, and, upon interaction with the proteasome core, it opens the proteasomal gate to allow substrate entry into the proteolytic chamber. Unlike Mpa and Cpa, Bpa has no ATPase activity and thus only allows unstructured or partially unfolded proteins to enter. Due to its recent discovery, the Cpa-proteasome complex is less well-studied and no substrates or recruitment mechanisms are known so far. However, Cpa was shown to play a role under carbon starvation conditions.

In the bacterial organisms where the proteasome was first studied, it was found to be non-essential for normal growth, and natural substrates of proteasomal degradation were slow to be discovered. However, the field received new impetus from the observation that the persistence of the human pathogen *Mycobacterium tuberculosis* in host macrophages is supported by proteasomal degradation (Darwin et al., 2003; Gandotra et al., 2007). Remarkably, it was later discovered in this context that bacteria have evolved their own brand of protein modification analogous to ubiquitination to target substrates for proteasomal degradation (Pearce et al., 2008; Burns et al., 2009). The 20S proteasome in complex with ARC, referred to as Mpa (mycobacterial proteasomal ATPase) in mycobacteria, was found to degrade proteins covalently modified on specific lysine side-chains with prokaryotic ubiquitin-like protein Pup (Pearce et al., 2008; Striebel et al., 2010). Intriguingly, unlike the homologous degradation machinery, this modification pathway referred to as pupylation is genetically unrelated to eukaryotic ubiquitination and represents an example of convergent evolution (Iyer et al., 2008; Striebel et al., 2009; Özcelik et al., 2012). The modification enzymes are expressed from the same gene locus as the proteasomal subunit genes and the proteasomal ATPase, together forming the so-called Pup-proteasome system (PPS) gene locus (Figure 1).

However, pupylation as well as proteasomal degradation were suggested early on to also function in separate contexts. Firstly, about half of the sequenced actinobacterial organisms have lost the proteasome core-subunit genes from the PPS locus, retaining the pupylation enzymes. Furthermore, disruption of pupylation or proteasomal degradation did not generate identical phenotypes (Darwin et al., 2003; Gandotra et al., 2007, 2010). In fact, more recently it has become apparent that the proteasome can act in degradation pathways independently of pupylation by associating with alternative regulator complexes. These alternative regulators are not encoded in the PPS gene locus, but reside elsewhere in the genome. One such regulator is the bacterial proteasome activator Bpa, an ATP-independent ring-shaped assembly that has been suggested to recognize substrates based on their conformational state (Delley et al., 2014; Jastrab et al., 2015) (Figure 1).

In eukaryotes, in addition to degradation of cytosolic proteins, the proteasome is involved in the degradation of ER-resident proteins via the ERAD (ER-associated degradation) pathway, where an AAA+ protein called Cdc48 (also known as p97 or VCP) cooperates with the proteasome [reviewed in (Wolf and Stolz, 2012)]. Mycobacteria and other actinobacteria harbor a homolog of this protein (Unciuleac et al., 2016; Ziemski et al., 2018). It was recently shown that Cdc48-like protein of actinobacteria (Cpa) can associate with the bacterial 20S proteasome *in vitro*, suggesting it might act in cooperation with the proteasome under certain conditions *in vivo* (Figure 1).

The ability of the 20S core particle to interact with diverse regulator complexes puts its degradation activity at the center of different proteolysis pathways employing varying substrate recognition determinants (Figure 1; Table 1). One common theme for proteasomal degradation that is emerging from the study of the various pathways, is that, although dispensable under standard culture conditions, it imparts a survival advantage under specific cellular conditions, amongst them nutrient starvation, exposure to damaging agents like nitric oxide and reactive oxygen species or exposure to heat shock.

**Table 1:** Comparison of the actinobacterial 20S proteasome-associated regulator complexes.

	**Mpa/ARC**	**Bpa**	**Cpa**
ATPase activity	Yes	No	Yes
Oligomeric state	Hexamer	Dodecamer	Hexamer
Relevant stress condition	Persistence, nitrogen starvation, DNA damage	Heat shock	Carbon starvation
20S core interaction	GxYx	GxYx	?
Substrate recruitment requirements	Pupylation, N-terminal coiled-coil of Mpa	Disorder	N-domain? Adapter proteins?
Known/well-studied substrates	e.g., Mpa, PanB, FabD, RecA, LOG	HspR	?

## Targeted Proteasomal Degradation

The power of using a dedicated targeting system for recruitment to proteasomal degradation lies in the ability to render a very large and diverse range of proteins as degradation substrates in a selective and temporally controlled manner. Furthermore, the targeting pathway itself can be specifically regulated and fine-tuned to the cellular needs. Eukaryotes have perfected this mode of substrate recruitment in form of the multilayered protein ubiquitination cascade (Metzger et al., 2012). Pupylation, the covalent attachment of the small, intrinsically disordered protein Pup to the substrate protein by formation of an isopeptide bond between Pup's C-terminal glutamyl moiety and a lysine on the substrate protein, is catalyzed by a single ligase (PafA, proteasome accessory factor A) (Pearce et al., 2008; Striebel et al., 2009). The formation of the isopeptide bond through the γ-carboxylate rather than the α-carboxylate bespeaks the ancestry of the Pup ligase (Sutter et al., 2010). It belongs to the family of γ-glutamyl-amine ligases and likely descends from an ancient glutamine synthetase enzyme (Iyer et al., 2008). Akin to the reaction mechanism of glutamine synthetase, Pup ligase PafA catalyzes a two-step reaction proceeding through a γ-glutamyl phosphate-mixed anhydride intermediate that is nucleophilically attacked by the ε-amino group of the substrate lysine (Guth et al., 2011). Interestingly, the PPS locus encodes a homolog of PafA that likely arose originally by gene duplication, and has evolved into a depupylase enzyme catalyzing the cleavage of the glutaminyl-C-N bond (Burns et al., 2010; Imkamp et al., 2010). In mycobacteria, depupylase Dop (deamidase of Pup) has in addition adopted a further regulatory role on the pupylation pathway, since Pup is encoded with a C-terminal glutamine residue that first has to be deamidated by Dop to a glutamate before PafA can carry out the pupylation (Striebel et al., 2009). This convoluted reaction circuit demonstrates the many levels of control that can be exerted through this recruitment pathway.

Pup mediates the recognition of pupylated substrates at the proteasomal ATPase Mpa (Pearce et al., 2008). In the eukaryotic 19S regulatory particle, the N-terminal coiled-coil domains of the regulatory particle ATPases (Rpts) mediate interactions with non-ATPase components of the 19S complex (Ler et al., 2012). In the architecturally homologous Mpa, the N-terminal coiled-coil domains serve as binding platform for Pup (Sutter et al., 2009; Wang et al., 2010). Upon binding to Mpa, Pup undergoes a disorder-to-order transition forming a single helix from the last two thirds of its primary sequence and a shared, antiparallel coiled-coil with Mpa's N-terminal domains, thereby pointing the still disordered N-terminal region of Pup directly into the translocation pore. The disordered N-terminus of Pup has therefore been suggested to serve as a threading handle allowing the ATPase-driven pore loop movements of Mpa to initially pull Pup and subsequently the substrate through the Mpa pore, extruding the unraveled polypeptide into the proteasome chamber. It is interesting in this context that some components of the PPS locus, the proteasomal ATPase Mpa and the Pup ligase PafA are themselves targets of pupylation, further underlining the intricate regulatory circuits involved in this proteasomal degradation pathway (Delley et al., 2012; Chen et al., 2016).

Analogous to the eukaryotic and archaeal proteasome, interaction of Mpa with the proteasomal core is mediated by Mpa's C-terminal tails featuring a conserved GxYx motif (Smith et al., 2007; Rabl et al., 2008; Striebel et al., 2010). Similarly, these tails are thought to insert into binding pockets on the proteasome α-ring surface, each formed by two adjacent subunits. Despite being a required feature, the GxYx motif in Mpa is however not sufficient *in vitro* to support a stable interaction with the core particle and allow efficient protein degradation (Wang et al., 2009; Striebel et al., 2010). Researchers relied on the use of a proteasome core variant for *in vitro* studies, in which the α-subunits are truncated N-terminally by seven or eight residues (Lin et al., 2006; Wang et al., 2009; Striebel et al., 2010). It is still unclear how a stably interacting Mpa-proteasome complex is maintained *in vivo*.

Proteomic studies have identified hundreds of proteins of diverse fold, size and oligomeric states, associated with a wide range of cellular functions as pupylation targets (Festa et al., 2010; Poulsen et al., 2010; Watrous et al., 2010; Yun et al., 2012; Küberl et al., 2014; Boubakri et al., 2015; Compton et al., 2015; Müller et al., 2018). It is therefore not surprising that Pup-driven degradation appears to serve multifaceted roles *in vivo*. Most prominently, in a murine Mtb infection model, silencing of the proteasome decreases bacterial counts and deletion of *pafA* significantly increases survival of the infected mice (Gandotra et al., 2007). Persistence of Mtb inside host macrophages was traced back to prevention of toxic effects caused by nitric oxide (NO) produced by the macrophage (Darwin et al., 2003; Samanovic et al., 2015). Paradoxically, breakdown products of cytokinins produced by Mtb itself cause a strongly bacteriotoxic effect together with NO, requiring the rapid, Pup-mediated removal of the cytokinin-producing Mtb enzyme following infection. Apparently, a significant advantage for survival is in this case gained by degradation of an individual proteasomal substrate. The PPS seems to be closely connected to nitrogen metabolism. Disruption of pupylation impairs survival of *M. smegmatis* in medium lacking nitrogen sources (Elharar et al., 2014). It was hypothesized that Pup-driven proteasomal degradation in this scenario might contribute to amino acid recycling to provide nitrogenous biosynthesis precursors. However, another study suggested a direct link to the assimilation of nitrogen, as key proteins involved in this process showed significantly changed levels in a *pup* knockout strain (Fascellaro et al., 2016). Recent results have also provided a link between the PPS gene locus and the mycobacterial DNA damage response (Fudrini Olivencia et al., 2017; Müller et al., 2018). It was discovered that PafBC, a protein complex encoded in an operon together with Pup ligase PafA in Mtb, is the master activator of the DNA damage response in mycobacteria, where it activates transcription of 150 genes involved in DNA repair and the oxidative stress response. Notably, PafBC also affects plasmid copy numbers, which has been interpreted as *pafA* regulation (Korman et al., 2018). However, previous studies did not observe an influence of *pafBC* deletion on *pafA* transcript levels, or binding of PafBC to the *pafA* promoter region *in vivo* (Fudrini Olivencia et al., 2017; Müller et al., 2018). Pupylation followed by proteasomal degradation helps maintain a temporally controlled stress response by removing many of the proteins upregulated during DNA damage stress.

## Proteasome as a Protein Stress Sensor

An entirely different mode of substrate recruitment to proteasomal degradation was discovered more recently with the identification of an ATP-independent, ring-shaped activator complex termed Bpa (bacterial proteasome activator, also referred to as PafE) (Delley et al., 2014; Jastrab et al., 2015). Bpa employs the same C-terminal GxYx motif as Mpa to dock into binding pockets located between the α-ring subunits of the 20S particle. The C-terminal carboxylate of the regulator forms a salt-bridge with a conserved lysine at the bottom of the proteasomal binding pocket and the conserved penultimate tyrosine stacks with a conserved arginine in the docking site (Bolten et al., 2016).

However, while Mpa is a multi-domain AAA+ protein (Wang et al., 2009), Bpa is a small, single-domain four-helix bundle lacking any ATP-binding or ATP-hydrolyzing activities. The Bpa protomers arrange into a twelve-membered ring with a 40 Å-wide pore, offering up a wide platform for substrate protein binding (Bai et al., 2016; Bolten et al., 2016). Interaction of Bpa with the proteasome opens the proteasomal α-ring gate, leading to an open conduit through the Bpa-ring pore into the 20S degradation chamber. Yet, due to its inability to use ATP hydrolysis for driving substrate-unfolding and translocation, Bpa, unlike Mpa, cannot mediate the degradation of stably folded protein substrates. Instead, Bpa was found to enhance the degradation of the unstructured model substrate β-casein, suggesting that recruitment to the Bpa-mediated proteasomal degradation pathway involves conformational disorder as a substrate determinant.

Indeed, heat shock repressor HspR was identified as the first natural degradation substrate of this pathway (Jastrab et al., 2015). HspR represses the expression of Hsp70 and ClpB, two chaperones involved in protein quality control (Bucca et al., 2000). This link to the heat-shock response as well as the fact that the *bpa* knockout strain shows a heat-shock sensitive phenotype (Jastrab et al., 2015) further supports the notion that the Bpa-proteasome degradation pathway is important under stress conditions, where the conformation of individual proteins or protein domains might be compromised. While substrates would have to enter the proteasome chamber by passive diffusion, the Bpa ring could provide a binding surface that increases the residence time of (partially) unstructured proteins proximal to the open 20S pore, thereby increasing the likelihood of substrate diffusing into the proteasome chamber.

## Cdc48-like Protein of Actinobacteria

In eukaryotes, in order to be degraded, ER-resident proteins have to be retrotranslocated out of the ER into the cytoplasm, where the 26S proteasome is located (Ruggiano et al., 2014). In this degradation pathway called ER-associated degradation (ERAD), the proteasome collaborates with the AAA+ protein Cdc48 (also known as p97 or VCP) (Wolf and Stolz, 2012). Several recent studies in eukaryotes and archaea have suggested that Cdc48 can directly bind to the 20S cylinder forming a Cdc48-20S proteasome complex (Barthelme and Sauer, 2012; Barthelme et al., 2015; Esaki et al., 2018).

Mycobacteria and many other actinobacteria possess a homolog of Cdc48 referred to as Cpa (Cdc48-like protein of actinobacteria) (Unciuleac et al., 2016; Ziemski et al., 2018). Cpa is encoded outside the PPS gene locus, but co-occurrence with the proteasome exists. All members of actinobacteria that encode Cdc48 also harbor the proteasomal subunits genes; although some actinobacteria exist that have the proteasome but not Cpa.

It has been demonstrated that Cpa can interact with the 20S core particle *in vitro*, forming ring-stacking, collinear complexes (Ziemski et al., 2018). However, unlike Mpa and Bpa, the C-termini of Cpa do not feature the penultimate tyrosine that mediates binding to the proteasomal α-ring pockets. A *cpa* deletion strain of *M. smegmatis* exhibits growth defects under carbon starvation and the levels of several hundred proteins are changed more than 3-fold (Ziemski et al., 2018). However, since multiple regulatory factors are amongst them and accumulation and depletion were observed in almost equal measure, no conclusions could be drawn about potential substrates of Cpa. Only the cluster of orthologous gene (COG) functional class J (translation/ribosomal structure and biogenesis) exhibited significantly more accumulated proteins than depleted proteins (20 vs. 7), potentially pointing toward a role in ribosome remodeling during starvation. Still, to date, no natural substrate proteins have been identified for Cpa and *in vitro* degradation of model substrates by Cpa in cooperation with the proteasome has also not been demonstrated. Further studies will be needed to establish a potential *in vivo* connection between Cpa and the 20S proteasome and to better understand the role of Cpa in mycobacteria.

## Conclusions

Actinobacteria have acquired a proteasome by horizontal gene transfer to expand their degradation repertoire beyond the canonical compartmentalizing proteases found in bacteria, like for example ClpXP and ClpCP proteases, or FtsH. Indeed, they have not only maintained the proteasome in their genome, but have evolved multiple substrate recruitment strategies converging on the proteasome as the unifying degradation module. The interaction with multiple regulators adds to the functional versatility of proteasomal degradation, and it offers the possibility of differential regulation of access for different substrate classes to proteasomal degradation.

One shared theme appears to be that these degradation pathways are important during stresses encountered by the bacteria, rendering them conditionally needed. Starvation for nutrients is a common occurrence in the natural environment of actinobacteria and requires adaptation of metabolic pathways as well as adjustment of translation activities under maintenance of minimal protein synthesis. Amino acid recycling, as was proposed for *M. smegmatis* under nitrogen starvation conditions (Elharar et al., 2014), might go hand-in-hand with a more specific role of degradation in adjusting the levels of nitrogen metabolism enzymes under these same conditions (Fascellaro et al., 2016). Partially disordered or damaged proteins on the other hand occur under toxic conditions like oxidative stress or heat shock. In this case, the Bpa-dependent degradation route might be involved both in promoting the heat-shock response via degradation of HspR, and indirectly removing conformationally compromised proteins.

From the range of conditions and cellular contexts in which proteasomal degradation appears to be involved, it is obvious that after its transfer into bacteria, the proteasome has adopted an ever-growing list of roles over the course of evolution. It can be expected that many more remain to be uncovered in the future.

## Author Contributions

AM and EW-B contributed to drafting, writing and editing of the manuscript.

### Conflict of Interest Statement

The authors declare that the research was conducted in the absence of any commercial or financial relationships that could be construed as a potential conflict of interest.
